# Promoting and achieving excellence in the delivery of Integrated Allergy Care: the European Academy of Allergy & Clinical Immunology competencies for allied health professionals working in allergy

**DOI:** 10.1186/s13601-018-0218-7

**Published:** 2018-08-21

**Authors:** I. J. Skypala, N. W. de Jong, E. Angier, J. Gardner, I. Kull, D. Ryan, C. Venter, B. J. Vlieg-Boerstra, K. Grimshaw

**Affiliations:** 1 Royal Brompton and Harefield NHS Foundation Trust, Sydney Street, London, SW3 6NP UK; 2 Imperial College, London, UK; 3 Erasmus MC, Rotterdam, The Netherlands; 4 University of Southampton, Southampton, UK; 5 Great North Children’s Hospital, Newcastle, UK; 6 Newcastle University, Newcastle, UK; 7 Karolinska Institutet, Stockholm, Sweden; 8 Sachs Children’s Hospital, Stockholm, Sweden; 9 Usher Institute, University of Edinburgh, Edinburgh, UK; 10 Denver School of Medicine, Colorado Children’s Hospital, University of Colorado, Denver, USA; 11 Onze Lieve Vrouwe Gasthuis (OLVG), Amsterdam, The Netherlands; 12 Southampton Children’s Hospital, Southampton, UK

**Keywords:** Allergy, Allied health professionals, Competency

## Abstract

The multi-disciplinary team approach is an effective model for patient care. Allied health professionals (AHPs) are an important part of such teams, bringing specific knowledge and skills related to the target patient population. The AHPs most often involved in allergy care are nurses and dietitians. Nurses are often involved in the care of patients with all types of allergy and also with asthma, whilst allergy-specialist dietitians provide vital nutritional and dietary support for the diagnosis and management of food allergy. There are many other AHPs who have a role to play in allergy care, including physiotherapists, psychologists, pharmacists and speech therapists, and their involvement is likely to develop as allergy care becomes more rooted in the community. With the development of multi-professional teams comes the requirement for disease-specific knowledge and skill sets, with all allergy team members required to have baseline knowledge and competency of the condition being managed. Whilst some competencies for AHPs practising in other disease states have been published, none are available for allergic disease against which AHPs can be benchmarked. The European Academy of Allergy & Clinical Immunology (EAACI) recognised this need, and supported the establishment of a Task Force to develop allergy-focussed competencies for AHPs. The varied skills, expertise and professional background of the Task Force members enabled the creation of a set of allergy competencies relevant to all AHPs working in allergy. It is recognised that the training and allergy expertise of AHPs, and their role within the allergy setting, will vary considerably depending on the country. However, it is important for patient care, that all AHP involved in allergy services have access to training, of a sufficiently high enough level to be aspirational and enable the continued growth and development of a wide range of allergy services, given the increasing need. The EAACI competencies will provide an important benchmark for allergy knowledge and skills against which education and training can be designed and health care professionals can subsequently be measured. However, more importantly, the EAACI AHP allergy competencies will enable the development and reach of specialist allergy services, with allergy-specialist AHPs undertaking key roles, especially in the community care setting.

**Electronic supplementary material:**

The online version of this article (10.1186/s13601-018-0218-7) contains supplementary material, which is available to authorized users.

## Background

Allied health professionals (AHPs) are increasingly working at the forefront of healthcare. The roles and responsibilities will vary depending on local availability and skills, but AHPs often have a high level of training, increasingly at post-graduate level. The prevalence of allergic diseases has in some countries been accompanied by an increasingly prominent role for AHPs such as nurses, dietitians, psychologists, physician assistants, pharmacists and others. It is well-recognised that patients may benefit from multi-disciplinary team input providing allergy care, so that the ever-increasing burden of allergy care provision can be shared, and the patient’s needs are addressed by the person with the most appropriate skill set. In Europe, the development and involvement of AHPs in allergy service provision varies according to the degree of training and accepted norms and requirements in individual countries [1]. In order to meet the growing demand for allergy services, it is important not only to make best use of available resources, but also to ensure continued quality and safety of service provision. Benchmarks, in the form of competencies, are a useful way of ensuring all AHPs working in allergy have the correct level of knowledge and skills, clear career pathways and support within the system, for staff retention and workforce planning.

## The multi-disciplinary team and the competent practitioner

Allergy is a complex multi-organ disease, coexisting with other organ-specific disorders that have a common allergic basis [2]. Most broad disease categories (e.g. asthma, rhinitis, anaphylaxis, dermatitis) may have both allergic and non-allergic components which in turn may overlap in the same individual and confound both diagnosis and management [3]. The popular hypothesis, known as the atopic march, proposes a set of sequential allergy and respiratory disorders developing in early childhood and contributing to the burden of disease in developed countries [4]. Allergic rhinitis, asthma, food allergy and eczema are common co-morbidities in both children and adults, with a significant change over time in the prevalence of each co-morbidity as children become adults [5–7]. Diagnosis and management is made even more complex when faced with patient beliefs and experiences, sometimes incurred as a result of misdiagnosis, which in itself may be as a consequence of using of invalid tests or the poor interpretation of history and tests by health care professionals [8]. The complexity of the condition demands a multidisciplinary team approach. A team has been described as being ‘a small number of consistent people committed to a relevant shared purpose, with common performance goals, complementary and overlapping skills, and a common approach to their work” [9]. The acronym TEAMWORK, stands for Team, Enthusiasm, Accessibility, Motivation, Workplace, Objectives, Role and Kinship [10] encompasses all of the requirements for harmonious working for the benefit of improving outcomes for the allergic patient. Team members should have equal status within the team including the principles of team based healthcare such as shared goals, clear roles, mutual trust, measurable outcomes, and clear communication which should also involve the patient as an equal member of the team [11].

One definition of integrated care is “to impose the patient perception as the organising principle of service delivery” [12] or more simply, placing the patient at the centre of care delivery. Working across professional boundaries, and encompassing ‘horizontal and vertical integration’, are some of the processes that can enable and support healthcare closer to the community and extended services [13, 14]. It has been demonstrated that well-structured teams and ‘cultures of engagement’ are related to decreased patient mortality and financial stability in health services [15]. However fundamental to the success of any model for team-based care is the skill and reliability of team members, with investment needed in order to support them to become and remain competent and expert [16]. The modern delivery of health care advocates the consideration as to whether the team member has the ‘competencies’ to deliver the care and not their ‘role or hierarchy’ of that person within the health system. A recent literature search on “Interdisciplinary Teamwork and Collaboration” and “Evidence-Based Practice” revealed these themes were largely identified in position papers linked to the skill level *competent and expert*, respectively [17]. Competencies recognise the importance of attaining and demonstrating both practical skills and a theoretical grounding in the subject. Competent allergy-specialist AHPs should enhance ‘workforce agility’ to improve the current provision of allergy care and support the patient journey. In addition to the “hard” skill and knowledge competencies, it is important for AHPs to also master the “softer” skills such as communication coupled with the ability to recognize and manage the patient’s own ideas concerns and expectations [18].

National and international professional associations, expert panels, consortia, centres, institutes and convened committees have published or sponsored position papers with recommendations for competencies essential for AHPs to provide high-quality, effective and safe patient care [19–24]. Published data suggests some AHPs feel they only have moderate proficiency in allergy management with a need to increase their knowledge [25, 26]. Education programmes have been shown to work well, but these need to be competency driven and based on evidence [27]. Such competencies may serve as the basis for design of curricula intended to educate diverse health professionals who subsequently demonstrate the knowledge, skills, and attitudes required to provide effective and safe allergy patient care [28].

## The role of AHPs in the diagnosis and management of allergic disease

### Nurses in asthma and allergy care

Specialist nurses are a valuable asset in chronic disease management, who, with appropriate training and competency assessment can provide a high standard of care [29]. Studies in asthma and other chronic diseases have shown positive outcomes for patients and improvement in the management of a patient’s condition with the support of a clinical nurse specialist [30]. Rance et al. noted how vital the specialist nurse role was in achieving and maintaining asthma control [31]. Another important area is patient training when prescribing auto-injectors with adrenaline for patients at risk of anaphylaxis, in order that they receive the correct training [32]. In addition, the nurse has also been shown to play an important role in the management of atopic dermatitis [33]. One of the nurse’s main tasks in the MDT is to provide patient-centred and holistic education and support for self-care. Nurse specialists in allergy often liaise between patient, physician and other healthcare professionals to optimise communication and ensure the patient fully understands their diagnosis and adheres to treatment. Many nurses across the world are involved in the implementation of precision medicine treatments for allergy and asthma, and also participate in or lead research into the management of allergic diseases. In some countries, nurses hold prescribing qualifications enabling them to take a more advanced role and supporting more patients to be seen. The development of the nurse’s role has, in countries such as the United Kingdom (UK) and Sweden, led to the development of competence criteria for asthma and allergy nurses. However, in many other countries, the development of the nurse specialist in allergy has happened in an ad hoc fashion. Consequently, there is a need to define competencies in order to standardise care and practice, especially since many patients with asthma are seen by nurses outside of the hospital setting [34].

### The allergy specialist dietitian

The allergy specialist dietitian has a role in the prevention, diagnosis and management of food allergy. The allergy-focussed diet history is the key to establishing or refuting a diagnosis of food allergy [35, 36]. This is one of the core competences of dietitians who are trained to evaluate dietary habits and collect detailed information about nutritional intake, foods habitually eaten, foods avoided, body mass index (BMI) and growth. The dietitian can thus not only analyse the diet history for the likely food trigger, she/he can also assess nutritional intake, nutritional status, growth in children or unintended weight loss in adults. Nutritional management is vital for food allergic children, who have lower intakes of total energy and macro/micronutrients, and are often smaller and lighter than their non-allergic counterparts [37, 38]. Conversely, the poor nutritional quality of exclusion diets may also result in obesity in the allergic paediatric population [39]. It has been shown that dietary counselling results in a significant improvement in the nutritional status of children with food allergy [40]. This is important since unlicensed nutritional practitioners and non-validated food allergy tests can place vulnerable allergic individuals at nutritional risk [41, 42]. Allergy specialist dietitians can also provide advice on the individualised avoidance of the specific food(s) reported or known to provoke allergic symptoms [43]. The prescription of an elimination diet is an important tool to ensure an accurate diagnosis is made and the correct management plan implemented. Since milk, egg, wheat and soy allergy often remit in late childhood, a dietitian can also assist in reviewing the continued need for the dietary exclusion and enable individualised re-introduction of those foods [44]. In addition to working with food allergic individuals, dietitians also play a role in providing information for the prevention and management of food allergy and other allergic conditions such as asthma and atopic dermatitis. Like nurses, they are also involved in and/or lead allergy research.

### Other allied health professionals

Psychologists, pharmacists, physiotherapists and speech and language therapists also deliver important aspects of care to the allergy patient. The psychologist empowers the allergy sufferer and their family by the application of coping strategies to support emotional wellbeing and the day to day management of their food allergy. Psychological interventions to support children with food allergies and their families have been shown to be significantly beneficial for both psychological and health outcomes [45, 46]. Pharmacists are frequently consulted regarding suitable medications for individual allergy patients which requires them to have a knowledge of what hypersensitivity reactions are, which drugs can be used to treat them, those drugs likely to cause hypersensitivity reactions, and which drugs are contraindicated for individuals who may require adrenaline to treat an allergic reaction [47]. Community pharmacists are often asked to provide advice on, or medications for, allergic conditions, which can allow them insight as to whether appropriate treatment has been prescribed, and ensure training is received on the correct use of adrenaline devices, nasal sprays and inhalers [32, 48, 49]. Many patients with food allergy are diagnosed in infancy. This early experience of an adverse reaction to food can often lead to feeding difficulties associated with taste, textures and an aversion to feeding utensils such as spoons, cups and bottles. Speech and Language therapists are vital in combatting these feeding difficulties but they need to have knowledge of food allergy in order to appropriately manage these infants/children. Respiratory therapists or physiotherapists have an important role to play in the management of asthma, and have been shown to improve outcomes and reduce the cost of care [50]. Paramedics and ambulance staff may be the first to assess and treat severe manifestations of an allergic reaction. Training AHP staff providing front-line emergency care will enhance and support the patient’s allergy journey.

## The development and overview of the EAACI AHPs competencies

There is no known international agreement as to the essential competencies across all stages of skill acquisition, for all AHPs working with the allergic patient. The AHPs Interest Group of the European Academy of Allergy & Clinical Immunology (EAACI) established a Task Force in 2014 to consider the role of the AHPs in the field of allergy, understand the differences between countries, the competencies required and what the educational needs are. The Task Force consisted of a number of highly experienced and expert AHPs working in allergy from different countries, and also physicians and General Practitioners with a special interest in allergy. Competencies for nurses new to allergy, which were based on Benner’s paper “From Novice to Expert” published by the British Society of Allergy & Clinical Immunology, were used as a basis for the development of the AHPs competencies [51, 52]. This nurses’ competency document template was enhanced to recognise other AHPs, retaining core principles but also adding elements to reflect the complexity of care and contribution of different AHPs groups.

The resulting EAACI AHPs competencies document (Additional file 1) provides a statement of the theoretical knowledge of allergic disease required by all AHPs working in allergy. The knowledge statement is accompanied by baseline competencies which all AHPs should possess, including basic concepts of the aetiology, diagnosis and management of allergic disease as well as an understanding of non-allergic conditions presenting in an allergy clinic. This “background” level of knowledge forms a framework onto which practitioners can pin their own specific knowledge and skill set for their particular role, such as dietary modification, drug allergy, or immunotherapy. The competencies are underpinned by the Global Atlas of Allergy [53], other EAACI-sponsored guidelines and other internationally accepted reference standards. They are divided into four different sections, the first being the symptoms and features of allergic and non-immune mediated disease. This section covers the knowledge and skills needed to understand the immune system, allergic diseases, epidemiology and risk factors, the allergens themselves, and non-allergic conditions presenting in an allergy clinic. The second section focusses on the diagnosis of allergic disease, including clinical history, tests and oral provocation. The third section covers the management of anaphylaxis, food allergy, drug allergy, immunotherapy, quality of life and individualised support for patients and their families. The final section reviews wider healthcare issues such as the evidence base for allergy, ethical issues and continuous professional development. Each section lists the knowledge and understanding required for each topic subheading, followed by the performance criteria.

## Conclusion

It is recognised that the training and allergy expertise of AHPs, and their role within the allergy setting, will vary considerably depending on the country. However, it is important for patient care, that all AHP involved in allergy services have access to training. As allergy can present in many ways to different services, it is essential to prepare and equip the diverse workforce with allergy competencies. Patients deserve good communication and timely care, which could be achieved by wider AHPs involvement. Additionally, an adaptive workforce can provide a sustainable solution to help meet the challenges of the increasing numbers of patients with suspected allergy and the concurrent shortage of specialists. This EAACI AHP Competency document paves the way for standardised quality allergy pathways that enhance the patient experience with good communication. It can also act as a basis for courses and training, and a reference for policymakers, raising awareness of the potential support and investment required for workforce remodelling and integrated care for allergy services. AHPs are in a ‘state of readiness’ to provide further extended services, improving public access to proficient, allergy-aware health care professionals which in turn should promote rapid diagnosis and seamless management for all those with suspected allergy across the age spectrum.

## Additional file

**Additional file 1.** Competencies for allied health professionals working in a clinical allergy setting.

## Abbreviations

AHP: allied health professional
EAACI: European Academy of Allergy & Clinical Immunology
MDT: multi-disciplinary team
BMI: body mass index
UK: United Kingdom
