# Supplementary material for: Promoting and achieving excellence in the delivery of Integrated Allergy Care: the European Academy of Allergy & Clinical Immunology competencies for allied health professionals working in allergy
Source: Clin Transl Allergy. 2018 Aug 21;8:31. doi: 10.1186/s13601-018-0218-7 (PMC6102852; doi:10.1186/s13601-018-0218-7)
Supplement: Supplementary file 1 — Additional file 1. Competencies for allied health professionals working in a clinical allergy setting. [file 13601_2018_218_MOESM1_ESM.docx]

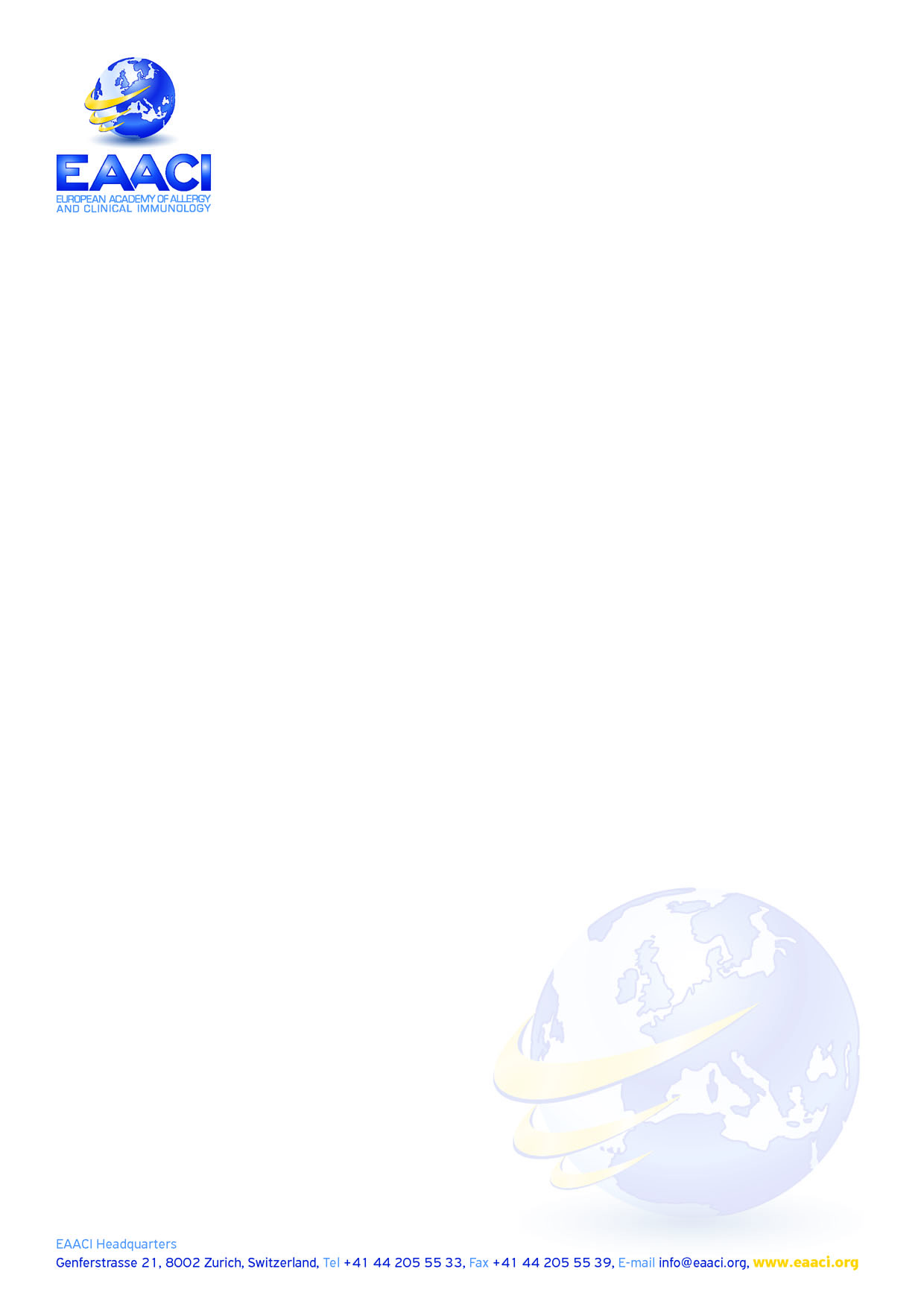


**EUROPEAN ACADEMY OF ALLERGY & CLINICAL IMMUNOLOGY**

**COMPETENCIES FOR ALLIED HEALTH PROFESSIONALS WORKING IN A CLINICAL ALLERGY SETTING**

**Contents Page**

| **Guideline for use** | **Page 3** |
| --- | --- |
| **Outline of Knowledge and Performance criteria** | **Page 4** |
| **Symptoms and features of allergic disease** | **Page 5** |
| **Diagnosis of allergic disease** | **Page 8** |
| **Management of allergic disease** | **Page 11** |
| **Wider healthcare issues** | **Page 15** |
| **References** | **Page 17** |
| **Appendix I – Task Force Membership** | **Page 20** |

**Guidelines for using the document**

The purpose of this document is to provide a statement of the theoretical knowledge of allergic disease required by all Allied Health professionals working in allergy including nurses, dietitians, nutritionists, physician’s assistants and psychologists.

The knowledge statement is accompanied by a framework of baseline competencies which can be adapted to suit the requirements of the individual practitioner. The competencies are underpinned by the Global Atlas of Allergy, EAACI-sponsored guidelines and other references

Verification of the acquisition of the quality standards outlined in this document, through annual appraisal or examination, would be desirable for all allied health practitioners who work with patients with a suspected or diagnosed allergic disease and their families.

**Knowledge and performance criteria:**

1. **The symptoms and features of allergic and non-immune mediated disease**

1.1 Basic knowledge of the immune system and how it operates, including Hypersensitivity Types I – IV and mast cells

1.2 Basic knowledge of allergic diseases, their symptoms and any grading systems, including anaphylaxis, eczema, urticaria,

rhinitis, asthma, and gastrointestinal disorders

1.3 Epidemiology and risk factors

1.4 Allergens (food, drug, insect venom, aeroallergens, contact allergens), seasonal distribution, cross reactivity

1.5 Basic knowledge of non-allergic conditions commonly presenting in an allergy clinic

1. **Diagnosis of allergic disease**

2.1 Taking and interpreting an allergy-focussed history

2.2 Skin prick, specific IgE tests and component tests

2.3 Respiratory and nasal tests

2.4 Other tests

2.5 Oral provocation tests for foods/drugs

1. **Management of allergic disease**

3.1 Management and recognition of acute severe allergic reactions (anaphylaxis and asthma)

3.2 Pharmacotherapy

3.3 Dietary modification

3.4 Drug Allergy

3.5 Immune therapy, indications and contra- indications

3.6 Impact of allergy on QOL

3.7 Providing individualised support and guidance

1. **Wider healthcare issues**

4.1 Evidence base

4.2 Ethical issues

4.3 Continuous professional development

**1) The symptoms and features of allergic disease and non-immune mediated conditions**

**1.1 Basic knowledge of the immune system and how it operates, including Hypersensitivity Types I – IV and mast cells**

| **KNOWLEDGE AND UNDERSTANDING:** |
| --- |
| 1. Understands terms frequently used to describe or reference allergy and allergic conditions |
| 2. Understands the process of sensitisation to IgE antibodies |
| 3. Understands the mechanism of an allergic response and the role of IgE |
| 4. Aware of the Gell and Coombes hypersensitivity classification Types 1-4 |
| **PERFORMANCE CRITERIA:** |
| 1. Can define atopy, sensitivity, hypersensitivity, sensitisation, allergy and anaphylaxis ^(1,2)^ |
| 2. Is able to outline the role of mast Cells, T Cells, B Cells, Eosinophils, Basophils and Dendritic Cells ^(1)^ |
| 3. Able to explain the process of sensitisation including the role of IgE ^(1)^ |
| 4. Able to outline the main effects of histamine release and relate this to allergy symptoms ^(1)^ |
| 5. Can give an example of the four types of hypersensitivity reactions as defined by Gell and Coombs - Immediate IgE mediated (type I), antibody-mediated cytotoxic reactions (type II), immune complex-mediated reactions (type III), and delayed type hypersensitivity (type IV) ^(1,3)^ |

**1.2 Basic knowledge of allergic diseases and their symptoms, including anaphylaxis, eczema, urticaria, rhinitis, asthma and gastrointestinal disorders, and any grading systems commonly employed**

| **KNOWLEDGE AND UNDERSTANDING** |
| --- |
| 1. Knowledge of the signs and symptoms of allergic reactions e.g. oropharyngeal, GI, skin, ophthalmic, upper and lower airway, systemic |
| 2. Awareness of commonly used grading systems for the different diseases |
| 3. Awareness of the nomenclature of allergic and non-allergic diseases presenting in the allergy clinic as defined by EAACI |
| 4. Awareness of the three different clinical criteria for identifying and recognising anaphylaxis |
| **PERFORMANCE CRITERIA:** |
| 1. Can cite nomenclature used to classify allergic diseases ^(1,2)^. |
| 2. Can cite the allergic rhinitis classification according to the ARIA guidelines (intermittent/persistent, mild, moderate and severe) and the recommended treatment for each category ^(4)^ |
| 3. Knows the names of the different scoring systems are for eczema (SCORAD/POEM) and what the first line treatment for eczema is ^(5,6)^ |
| 4. Ability to outline the main features of asthma and its management using appropriate guidelines e.g. ERS guidelines ^(7)^ GINA guidelines ^(8)^ |
| 5. Understanding of the usual presentations of urticaria according to the EAACI/GA2 LEN/EDF/WAO Guideline ^(9)^ |
| 6. Can quote the EAACI guidelines on anaphylaxis criteria for diagnosing anaphylaxis and emergency management first line interventions ^(10)^ |
| 7. Able to demonstrate using a structured approach to the diagnosis of IgE and non-IgE mediated food allergy and differential diagnoses which is supported by EAACI guidelines ^(11,12)^ |

**1.3 Epidemiology and risk factors of allergic and non-immune mediated disease**

| **KNOWLEDGE AND UNDERSTANDING** |
| --- |
| 1. Understanding of the concepts relating to the “Allergy Epidemic” |
| 2. Demonstrates a basic understanding of environmental risk factors including smoking and occupational factors |
| 3. Awareness of the risk factors for developing allergic diseases |
| 4. Is aware of the current lack of data on non IgE and non-immune mediated conditions |
| **PERFORMANCE CRITERIA:**   1. Can describe the natural history of allergy and the concept of the allergic march ^(1,13,14)^ |
| 1. Demonstrates a knowledge of risk factors including food, home environment and working environment ^(1,11,15,16,17)^ |
| 1. Able to describe how allergic sensitisation can lead to development of other allergic disease. ^(1, 13)^ |
| 1. Can demonstrate how epidemiological findings from population cohorts are utilised in day to day practice. ^(1)^ |

**1.4 Allergens (food, drug, insect venom, aeroallergens, contact allergens), seasonal distribution, cross reactivity**

| **KNOWLEDGE AND UNDERSTANDING** |
| --- |
| 1. Knowledge of which foods commonly cause food allergy and non-immune mediated reactions to foods in both children and adults |
| 2. Knowledge of which drugs are most frequently considered to provoke hypersensitivity reactions and which are contraindicated if adrenaline is required |
| 3. An awareness of venom allergy, specificity of insects involved and its presentation |
| 4. An understanding of aeroallergen sensitisation and allergy including seasonality, symptoms and cross reactivity with food allergy such as pollen food syndrome and mite/shellfish cross-reactions |
| 5. Knowledge of which contact allergens are most likely to provoke reactions in daily life and occupational situations |
| **PERFORMANCE CRITERIA:** |
| 1. Be able to describe the evidence and likely food triggers for immune and non-immune mediated food allergy, foods which commonly provoke reactions in childhood and those which normally present in adult life, and knowledge of which allergenic foods require to be labelled ^(1,12,18)^ |
| 2. Is able to cite which drug families are most likely to provoke hypersensitivity reactions e.g. Penicillin drugs ^(1, 19)^ |
| 3. Is able to cite common causes of venom allergy ^(20)^ |
| 4. Ability to cite the relevant aero-allergens and their clinical presentation. Also be able to describe the most common manifestations of cross-reactions between aeroallergens and foods ^(1,21)^ |
| 5. Be able to name some of the triggers of contact dermatitis and their clinical presentation ^(1, 22,23)^ |

**1.5 Basic knowledge of non-allergic conditions commonly presenting in an allergy clinic**

| **KNOWLEDGE AND UNDERSTANDING:** |
| --- |
| 1. Awareness of the differential diagnoses for the common allergic conditions presenting in an allergy clinic |
| **PERFORMANCE CRITERIA:** |
| 1. Can list likely differential diagnosis for food allergy e.g. coeliac disease, IBS, chronic spontaneous urticaria ^(1)^ |
| 1. Can list likely differential diagnosis for respiratory allergy e.g. hyperventilation syndrome, PCD, COPD, CF, bronchiectasis, non-allergic rhinitis and asthma (role specific) ^(7)^ |
| 1. Can list likely differential diagnosis for skin allergy e.g. scabies, dermatitis artefacta, pityrasis rosea, psoriasis ^(9)^ (role specific) |
| 4. Demonstrates an knowledge of non-allergic rhinitis and it’s causes e.g. viral, gustatory, drug induced (role-specific) ^(24)^ |

**2) Diagnosis and management of allergic disease**

**2.1 Taking and interpreting an allergy-focussed history**

| **KNOWLEDGE AND UNDERSTANDING** |
| --- |
| 1. Understand that a thorough clinical history is the cornerstone of the diagnosis and differential diagnosis of allergic disease |
| 1. Knowledge that there are standardised tools available e.g. EAACI diet history tools |
| 1. Know what information is required including the following: age, gender, ethnicity, presenting symptoms, triggers, personal medical history, family history, atopic history, co-factors, lifestyle, occupation, environmental influences, co-morbidities, medication, diet and nutrition |
| 4a. Food allergy  1. Knowledge of common allergenic foods for specific age groups  2. Knowledge of how to assess the diet to determine the likelihood of a food causing food allergy or other food-related symptoms  3. Knowledge of which foods may be cross-reacting with inhalant allergens  4. Use of the history to identify who is likely to be at risk of poor nutritional status  5. Awareness of which drugs and foods which can mimic allergic symptoms e.g. Beta blockers, ace inhibitors, scombroid poisoning, capsaicin sensitivity  6. An awareness of the relevance of co-factors such as exercise, alcohol, NSAIDS |
| 4b. Respiratory allergy  1. Knowledge of the common aeroallergens and their geographical differences  2. Awareness of occupational allergens  3. Awareness of the potential role of aspirin and NSAID in a reaction  4. Awareness of other drugs which may cause/exacerbate symptoms  5 Awareness of the co-existence of asthma and rhinitis |
| 4c. Skin allergy  1. Awareness of common contact allergens e.g. nickel, hair dye, lanolin, artificial henna, cosmetics, leather dyes  2. Awareness of other drugs which may cause/exacerbate symptoms  3. Awareness of the possible detrimental effects of the overuse of personal hygiene products |
| **PERFORMANCE CRITERIA:** |
| 1. Can explain why history is important in the diagnosis of allergy, including how it helps with the choice of diagnostic tests and what the symptoms and foods/drugs/aeroallergens/other triggers can indicate ^(11,21,25-27)^ |
| 2. Demonstrates a knowledge and ability to use and interpret standardised information gathering tools in order to plan further investigations as appropriate ^(11, 25)^ |
| 3. Can list all of the different criteria required for an allergy-focussed history ^(11,25,26)^ |
| 4a. Food Allergy - is able to demonstrate the interpretation of an allergy-focussed history in the diagnosis of food allergy using all the knowledge criteria ^(11,25,28-34)^ |
| 4b. Respiratory allergy - is able to demonstrate the interpretation of an allergy-focussed history in the diagnosis of respiratory allergy using all the knowledge criteria ^(7,35,36)^ |
| 4c. Skin allergy - is able to demonstrate the interpretation of an allergy-focussed history in the diagnosis of skin allergy using all the knowledge criteria ^(9)^ |

**2.2 Skin prick tests (SPT, prick to prick tests (PPT), specific IgE tests (SIgE) and component resolved diagnosis (CRD)**

| **KNOWLEDGE AND UNDERSTANDING:** |
| --- |
| 1. Understand the differences between SPT, PPT, SIgE and CRD, the predictive value of these tests and which test performs best for individual allergens within the area of practice |
| 2. Understand the concept, value, associated risks, contraindications and practical application of the SPT and PPT in allergy diagnosis |
| 3. Understand the concept, value, contraindications of the SIgE test and the difference between this test, SPT, PPT and CRD |
| 4. Knowledge of CRD, and which individual allergens are useful to test for in allergic diseases |
| 5. Knowledge of how to perform diagnostic tests, including consideration of shelf life and appropriate storage of test products, and how to interpret the test results using the best available evidence |
| **PERFORMANCE CRITERIA:** |
| 1. Able to describe all of the tests and specify the similarities and differences between them ^(12,21,34)^ |
| 2. Able to provide examples of when it is not appropriate to undertake SPT,PPT or SIgE ^(12)^ |
| 3. Able to interpret positive and negative test results and show understanding of their predictive value ^(12, 34)^ |
| 4. Can cite which are the main component allergens to test for in peanut allergy, Hazelnut allergy, resolution of milk allergy, resolution of egg allergy, pollen-food syndrome ^(35)^ |
| 5. Can perform an SPT or PPT according to EAACI guidelines ^(7, 36)^ |

**2.3 Respiratory and nasal tests - Peak flow, spirometry, nasal inspiratory tests, FeNO**

| **KNOWLEDGE AND UNDERSTANDING:** |
| --- |
| 1. Demonstrate an awareness of the different pulmonary function and/or inflammation e.g. reversibility testing, bronchial provocation tests, peak flow, spirometry, FeNO |
| 1. Knowledge of upper respiratory function tests e.g. peak nasal inspiratory flow, nasal provocation tests, nasal NO |
| 1. Understand the significance of peripheral eosinophilia |
| **PERFORMANCE CRITERIA:** |
| 1. Can prepare the patient, perform, interpret and evaluate pulmonary function tests (role-specific) ^(7,8)^ |

**2.4 Other tests**

| **KNOWLEDGE AND UNDERSTANDING:** |
| --- |
| 1. An awareness of less commonly used in-vitro/in-vivo diagnostic tests e.g. basophil activation test, intradermal testing, epicutaneous tests, patch testing |
| 2. Understanding of when patch testing may be helpful |
| 3. Knowledge of other non-validated tests for allergy and understand why they are not suitable |
| **PERFORMANCE CRITERIA:** |
| 1. Can describe how patch testing is carried out and what type of allergy diagnosis it can be used for ^(22,38)^ |
| 2. Has the ability to differentiate valid from non-valid tests for allergy and for IgG testing be able to give a rationale for why it is not a suitable test for allergy diagnosis ^(12,39)^ |
| 3. Describe the use of intradermal tests in drug and venom allergy (role specific) ^(26,40)^ |

**2.5 Oral provocation tests for foods/drugs**

| **KNOWLEDGE AND UNDERSTANDING:** |
| --- |
| 1. Knowledge of the different types of oral food challenge and how they are performed |
| 1. Awareness of the different types of drug allergy challenges and protocols |
| **PERFORMANCE CRITERIA:** |
| 1. Able to give an example of a protocol for an open oral food challenge schedule ^(12,41,42)^ |
| 1. Be able to describe a protocol for an open drug challenge (role specific) ^(26,27,43)^ |
| 1. Demonstrate a knowledge of local and national/international food/drug provocation guidelines appropriate to role ^(12,26,27,41-43)^ |

**3) Management of allergic disease**

**3.1 Management and recognition of acute severe allergic reactions (anaphylaxis and asthma)**

| **KNOWLEDGE AND UNDERSTANDING:** |
| --- |
| 1. Knowledge of basic life support skills |
| 1. Understanding of drugs used in the management of acute allergic reactions |
| 1. Knowledge of how to recognise the first signs of a severe allergic reaction and an awareness of the local acute allergic management plan |
| **PERFORMANCE CRITERIA: ^(1)^** |
| 1. An ability to apply the EAACI guidelines as a first line treatment for anaphylaxis ^(10)^ |
| 1. Be able to apply the relevant guidelines for first line treatment of rhinitis, asthma, food allergy and urticaria (see also section 1) ^(1-12)^ |
| 1. Provide evidence of recent formal basic life support training (within 2 years) |

**3.2 Pharmacotherapy**

| **KNOWLEDGE AND UNDERSTANDING:** |
| --- |
| 1. Knowledge of the techniques for the administration of inhaled medications for nasal/pulmonary allergy |
| 1. A basic knowledge of H1 and H2 antihistamines and their appropriate use in allergic disease |
| 1. A basic knowledge of topical treatments and application |
| 1. Knowledge of adrenaline use, including dose, route of administration, storage and expiry date |
| **PERFORMANCE CRITERIA:** |
| 1. An ability to describe the medications used in the treatment of allergic disease ^(1-12,44,45)^ |
| 1. Is able to report on the dosage, storage, correct method of administration of medications used in the treatment of allergic disease (role specific) |
| 1. Can demonstrate inhaler technique for a variety of inhalers against specified criteria (role specific) |
| 1. Can demonstrate how and when to use an adrenaline auto-injector |

**3.3. Dietary modification (role specific)**

| **KNOWLEDGE AND UNDERSTANDING:** |
| --- |
| 1. An understanding that an appropriate nutritionally sound elimination diet is the key treatment in food allergy |
| 1. Knowledge of how to apply diagnostic information to guide the individual prescription of appropriate dietary elimination and review (role specific) |
| 1. An awareness that dietary elimination can lead to nutritional inadequacy and individuals on long standing elimination diets, or avoiding staple foods are at highest risk for this, particularly if children |
| 1. A basic knowledge of the role of dietary replacement products and food labelling regulations |
| 1. In paediatrics, a knowledge of suitable milk substitutes for infants and pre-school children, their indications and contra-indications (role specific) |
| **PERFORMANCE CRITERIA:** |
| 1. Can construct, implement and re-evaluate an appropriate, nutritionally sound elimination diet in the treatment in food allergy (role specific) ^(28, 46-51)^ |
| 1. Demonstrates communication with members of the MDT as appropriate for to individuals requiring specialist nutritional and/or dietary intervention |
| 1. Can monitor growth in children and unintentional weight changes in adults ^(52-54)^ |
| 1. In paediatrics, can provide advice on suitable milk substitutes for infants and pre-school children, their indications and contra-indications ^(55,56)^ |

**3.4 Drug Allergy (role specific)**

| **KNOWLEDGE AND UNDERSTANDING:** |
| --- |
| 1. Knows the different drug reactions divided in Type A, B, C, D, E and F and is able to give examples. |
| 2. Knows that drug- induced skin Diseases are categorised in type I,II,III,and IV, and is able to give examples e.g. direct and delayed type. |
| 3. Knows that drug type IV reactions are divided in Type IV a,b,c,d |
| 4. Knows the risks of cross reactivity between drugs e.g. the B-lactam group and NSAIDs. |
| 5. Knows which diagnostic tests are used to confirm the specific drug allergy and which are contra-indicated. |
| 6. Knows that in certain cases desensitization with drugs is possible. |
| **PERFORMANCE CRITERIA:** |
| Is able to recognise adverse drug reactions in patients ^(26)^ |
| Is able to perform intracutaneous tests, prick tests, epicutaneous tests and challenges with drugs ^(26)^ |
| Is able to interpret skin tests with drugs e.g. false negative and false positive reactions ^(26)^ |

**3.5 Immune therapy, indications and contra- indications (role-specific)**

| **KNOWLEDGE AND UNDERSTANDING:** |
| --- |
| 1. Knows the clinical indications for AIT, commonly used routes of administration (SCIT and SLIT), and allergens involved. |
| 1. An understanding that AIT is contra- indicated in some circumstances |
| 1. An awareness of local/national/international guidelines on how to perform injections as appropriate |
| 4 An awareness of new routes of immunotherapy e.g. intra lymphatic |
| **PERFORMANCE CRITERIA:** |
| 1. Can demonstrate an awareness of how to administer AIT (role specific) ^(58-61)^ |
| 1. Is able to recognize patients at a high risk of an adverse reaction during AIT e.g. venom AIT (role specific) ^(58-61)^ |

**3.6 Impact of allergic diseases on QOL**

| **KNOWLEDGE AND UNDERSTANDING:** |
| --- |
| 1. An understanding of the impact of allergic disease on the individual and their family |
| 1. An understanding that there is a range of validated disease-specific quality of life and generic tools for use in food allergy management |
| **PERFORMANCE CRITERIA: ^(62-66)^** |
| 1. An ability to use health-related quality of life questionnaires appropriately (role specific) |
| 1. Demonstration that an individual’s quality of life and the impact of the diagnosis is considered when making allergy care recommendations |

**3.7 Providing individualised support and guidance**

| **KNOWLEDGE AND UNDERSTANDING:** |
| --- |
| 1. Have an awareness of the roles of the multi-disciplinary team (MDT) |
| 1. An awareness that a person-centred approach involves patient choice when planning, developing and accessing care |
| 1. An understanding that all patients with a diagnosed allergy require one to one advice specific to their individual needs and lifestyle |
| 1. An awareness that all patients need an individual risk assessment and written management plan |
| 1. Knowledge of the different types of support offered by patient support groups (See below) |
| 1. Knowledge of local, national, European and international support groups (See below) |
| 1. An understanding of which patients may need medical identification tags |
| **PERFORMANCE CRITERIA: ^(67)^** |
| 1. Involves the MDT as appropriate |
| 1. Able to create a self-management plan in conjunction with the patient and be able to communicate it at an appropriate level |
| 1. Able to advise on available and appropriate sources of support |
| 1. Able to discuss avoidance measures relevant to the diagnosis of the individual patient |

**4) Wider healthcare issues**

**4.1 Evidence base**

| **KNOWLEDGE AND UNDERSTANDING:** |
| --- |
| 1. Understanding of the importance of evidence-based practice as defined by Sackett ^(68)^: ‘Conscientious and explicit use of current best evidence in making decisions about the care of individual patients’ |
| 1. Knowledge of how to evaluate and assess evidence, including original research and review articles |
| 1. Awareness that clinical decisions should be underpinned by an efficient and thorough search of the literature, followed by a critical appraisal of the evidence and consideration of guidelines in the context of the individual patient |
| 1. Knowledge of how to identify and use the evidence base which support the development of protocols and the evaluation of existing resources |
| **PERFORMANCE CRITERIA: ^(69,70)^** |
| 1. Demonstrate ability to use the evidence base to develop protocols and/or care pathways to support practice within the MDT |
| 1. Evidence of involvement in the formulation of evidence-based allergy care |
| 1. Demonstrates consideration of the relevance and implementation of new guidelines as required |

**4.2 Ethical issues**

| **KNOWLEDGE AND UNDERSTANDING:** |
| --- |
| 1. An awareness of the importance of ethical issues in the delivery of allergy care |
| 1. Knowledge of care delivery in a manner that preserves and protects healthcare consumer autonomy, dignity, rights and beliefs |
| 1. Knowledge of confidentiality issues within legal and regulatory parameters |
| 1. Awareness of the need to continually assess and/or question healthcare practice for safety and quality improvement |
| 1. Knowledge of how to maintain a therapeutic relationship within appropriate professional role boundaries. |
| **PERFORMANCE CRITERIA: ^(71,72)^** |
| 1. Demonstrates the ability to obtain informed consent. |
| 1. Can demonstrate how to maintain patient confidentiality |
| 1. Demonstrates an understanding of what constitutes appropriate professional behaviour in a clinical relationship |
| 1. Demonstrates an understanding of ethical issues in the delivery of allergy care |

**4.3 Continuous professional development**

| **KNOWLEDGE AND UNDERSTANDING:** |
| --- |
| 1. An understanding of the need for a commitment to lifelong learning for optimal delivery of patient care |
| 1. Awareness of on-going educational activities related to appropriate knowledge bases and professional issues |
| 1. Understand the need to maintain the knowledge and skills required for an allergy specialist practitioner |
| 1. Understanding of the need to share educational findings, experiences, and ideas with peers in order to facilitate two-way learning |
| **PERFORMANCE CRITERIA: ^73)^** |
| 1. Is able to provide evidence of continual professional development |
| 1. Demonstrates the ability to incorporate appropriate research findings into everyday practice |
| 1. Demonstrates a commitment to lifelong learning through self-reflection and maintenance of professional records |
| 1. Seeks out formal and independent learning activities to maintain and develop clinical and professional skills and knowledge |

**References**

1. European Academy of Allergy & Clinical; Immunology. The Global Atlas of Allergy. <http://www.eaaci.org/globalatlas/GlobalAtlasAllergy.pdf>
2. Johansson, S.G., Bieber, T., Dahl, R., Friedmann, P.S., Lanier, B.Q., Lockey, R.F. et al 2004. Revised nomenclature for allergy for global use: Report of the Nomenclature Review Committee of the World Allergy Organization, October 2003. J Allergy Clin Immunol, 113, (5) 832-836
3. Coombs RRA, Gell PGH. The classification of allergic reactions underlying disease. In: Gell PGH and Coombs RRA (eds) Clinical Aspects of Immunology. 1963 pp317-337. Blackwell Scientific Publications. Oxford.
4. Brozek JL, Bousquet J, Baena-Cagnani CE, Bonini S, Canonica GW, Allergic Rhinitis and its Impact on Asthma (ARIA) guidelines: 2010 revision. J Allergy Clin Immunol. 2010;126:466-76. <http://www.ncbi.nlm.nih.gov/pubmed/20816182>
5. Severity scoring of atopic dermatitis: the SCORAD index. Consensus Report of the European Task Force on Atopic Dermatitis. Dermatology. 1993;186(1):23-31. <http://www.ncbi.nlm.nih.gov/pubmed/8435513>
6. Spuls PI et al. Patient-Oriented Eczema Measure (POEM), a core instrument to measure symptoms in clinical trials: a Harmonising Outcome Measures for Eczema (HOME) statement. Br J Dermatol. 2017 Apr;176(4):979-984.
7. Chung KF, Wenzel SE, Brozek JL, Bush A, Castro M, Sterk PJ et al. International ERS/ATS guidelines on definition, evaluation and treatment of severe asthma. Eur Respir J. 2014;43(2):343-373.
8. Global Initiative for Asthma. Global Strategy for Asthma Management and Prevention, 2017. [www.ginasthma.org](http://www.ginasthma.org)
9. Zuberbier T, Aberer W, Asero R, Bindslev-Jensen C, Brzoza Z, Canonica GW et al. The EAACI/GA(2) LEN/EDF/WAO Guideline for the definition, classification, diagnosis, and management of urticaria: the 2013 revision and update. Allergy. 2014;69(7):868-887.
10. Muraro A, Roberts G, Worm M, Bilò MB, Brockow K, et al. Anaphylaxis: guidelines from the European Academy of Allergy and Clinical Immunology. Allergy. 2014;69:1026-45 <http://www.ncbi.nlm.nih.gov/pubmed/24909803>
11. Muraro A, Agache I, Clark A, Sheikh A, Roberts G, Akdis CA et al. EAACI food allergy and anaphylaxis guidelines: managing patients with food allergy in the community. Allergy. 2014;69(8):1046-1057
12. Muraro A, Werfel T, Hoffmann-Sommergruber K, Roberts G, Beyer K et al. EAACI food allergy and anaphylaxis guidelines: diagnosis and management of food allergy. Allergy. 2014;69(5):1008-1025.http://www.ncbi.nlm.nih.gov/pubmed/24909706
13. Zheng T, et al. (2011). The Atopic March: Progression from Atopic Dermatitis to Allergic Rhinitis and Asthma. Allergy, Asthma and Immunology Research. 3(2), 67-73.
14. Thomsen SF. Epidemiology and natural history of atopic diseases. Eur Clin Respir J. 2015 Mar 24;2.
15. Grimshaw K, Logan K, O'Donovan S, Kiely M, Patient K, van Bilsen J et al. G. Modifying the infant's diet to prevent food allergy. Arch Dis Child. 2017;102(2):179-186.
16. Siracusa A, Folletti I, Gerth van Wijk R, Jeebhay MF, Moscato G, Quirce S, Raulf M, Rueff F, Walusiak-Skorupa J, Whitaker P, Tarlo SM. Occupational anaphylaxis – an EAACI task force consensus statement. Allergy 2015; 70: 141–152.
17. Raulf M, Buters J, Chapman M, Cecchi L, de Blay F, Doekes G, Eduard W, Heederik D, Jeebhay MF, Kespohl S, Krop E, Moscato G, Pala G, Quirce S, Sander I, Schlunssen V, Sigsgaard T, Walusiak-Skorupa J, Wiszniewska M, Wouters IM, Annesi-Maesano I. Monitoring of occupational and environmental aeroallergens – EAACI Position Paper. Allergy 2014; 69: 1280–1299.
18. European Union. (2007) Directive 2007/68/EC of the European Parliament amendment of Directive 2000/13/EC. OJL310: 11
19. Demoly P, Adkinson NF, Brockow K, Castells M, Chiriac AM, Greenberger PA, Khan DA, Lang DM, Park H-S, Pichler W, Sanchez-Borges M, Shiohara T, Thong BY-H. International Consensus on drug allergy. Allergy 2014; 69: 420–437
20. Sturm GJ, Varga EM, Roberts G, Mosbech H, Bilò MB, Akdis CA et al. EAACI Guidelines on Allergen Immunotherapy: Hymenoptera venom allergy. Allergy. 2017 Jul 27. doi: 10.1111/all.13262. [Epub ahead of print]PubMed PMID: 28748641.
21. Werfel T, Asero R, Ballmer-Weber BK, Beyer K, Enrique E, Knulst AC et al. Position paper of the EAACI: food allergy due to immunological cross-reactions with common inhalant allergens. Allergy 2015;70(9):1079–1090.
22. de Waard-van der Spek FB, Andersen KE, Darsow U, Mortz CG, Orton D et al. Allergic contact dermatitis in children: which factors are relevant? (review of the literature). Pediatr Allergy Immunol 2013: 24: 321–329.
23. Fonacier L, Bernstein DI, Pacheco K, Holness DL, Blessing-Moore J et al. Contact dermatitis: a practice parameter-update 2015. J Allergy Clin Immunol Pract. 2015 May-Jun;3(3 Suppl):S1-39
24. Scadding GK, Durham SR, Mirakian R, Jones NS, Leech SC, Farooque S et al. BSACI guidelines for the management of allergic and non-allergic rhinitis. Clin Exp Allergy. 2008;38(1):19-42.
25. Skypala IJ, Venter C, Meyer R, deJong NW, Fox AT, Groetch M et al. Allergy-focussed Diet History Task Force of the European Academy of Allergy and Clinical Immunology. The development of a standardised diet history tool to support the diagnosis of food allergy. Clin Transl Allergy. 2015 Feb 19;5:7.
26. Mayorga C, Celik G, Rouzaire P, Whitaker P, Bonadonna P, Rodrigues-Cernadas J et al.In vitro tests for Drug Allergy Task Force of EAACI Drug Interest Group. In vitro tests for drug hypersensitivity reactions: an ENDA/EAACI Drug Allergy Interest Group position paper. Allergy. 2016;71:1103-34.
27. Bonadonna P, Pagani M, Aberer W, Bilò MB, Brockow K, Oude Elberink H, GarveyL, Mosbech H, Romano A, Zanotti R, Torres MJ. Drug hypersensitivity in clonal mast cell disorders: ENDA/EAACI position paper. Allergy. 2015;70:755-63. Review.
28. Venter C, Laitinen K, Vlieg-Boerstra BJ. Nutritional aspects in diagnosis and management of food hypersensitivity-the dietitians role. Special issue Food hypersensitivity. J Allergy (Cairo). 2012;2012:269376
29. Nwaru BI, Hickstein L, Panesar SS, Roberts G, Muraro A, Sheikh A; EAACI Food Allergy and Anaphylaxis Guidelines Group. Prevalence of common food allergies in Europe: a systematic review and meta-analysis. Allergy. 2014;69:992-1007.
30. Bohle B,. Allergens and crossreactivity. Global atlas of Allergy. Akdis CA, AGache I. EAACI 2014.
31. Ferreira F, Gadermaier G, Wallner M. Tree pollen allergens. Global atlas of Allergy. Akdis CA, AGache I. EAACI 2014.
32. Ballmer-Weber B. Food allergens. Global atlas of Allergy. Akdis CA, AGache I. EAACI 2014.
33. Hoffman-Sommergruber K. Emerging allergens. Global atlas of Allergy. Akdis CA, Agache I. EAACI 2014.
34. Roberts G, Ollert M, Aalberse R, Austin M, Custovic A, DunnGalvin A et al. A new framework for the interpretation of IgE sensitization tests. Allergy. 2016;71(11):1540-1551.
35. EAACI Molecular Allergology Users Guide. EAACI 2016
36. Bousquet J, Heinzerling L, Bachert C, Papadopoulos NG, Bousquet PJ, Burney PG et al. Global Allergy and Asthma European Network.; Allergic Rhinitis and its Impact on Asthma.. Practical guide to skin prick tests in allergy to aeroallergens. Allergy. 2012;67(1):18-24.
37. Wallace DV, Dykewicz MS, Bernstein DI, Blessing-Moore J, Cox L, Khan DA, et al. The diagnosis and management of rhinitis: an updated practice parameter. J Allergy Clin Immunol 2008;122:S1-84.
38. Johansen JD, Aalto-Korte K, Agner T, Andersen KE, Bircher A, Bruze M et al. European Society of Contact Dermatitis guideline for diagnostic patch testing - recommendations on best practice. Contact Dermatitis. 2015;73(4):195-221.
39. Stapel SO, Asero R, Ballmer-Weber BK, Knol EF, Strobel S, Vieths S, Kleine-Tebbe J; EAACI Task Force. Testing for IgG4 against foods is not recommended as a diagnostic tool: EAACI Task Force Report. Allergy. 2008;63(7):793-796.
40. Brockow K, Romano A, Blanca M, Ring J, Pichler W, Demoly P. General considerations for skin test procedures in the diagnosis of drug hypersensitivity. Allergy. 2002 Jan;57(1):45-51.
41. Sampson HA, Gerth van Wijk R, Bindslev-Jensen C, Sicherer S, Teuber SS, Burks AW, et al. Standardizing double-blind, placebo-controlled oral food challenges: American Academy of Allergy, Asthma & Immunology-European Academy of Allergy and Clinical Immunology PRACTALL consensus report. J Allergy Clin Immunol 2012;130:1260-74.
42. Eigenmann P. In-vivo allergy diagnosis. Food provocation tests. Global atlas of Allergy. Akdis CA, AGache I. EAACI 2014.
43. Aberer W, Bircher A, Romano A, BlancaM, Campi P, Fernandez J et al. Drug provocation testing in the diagnosis of drug hypersensitivity reactions: general considerations. Allergy 2003;58:854–863
44. Bousquet J, Schünemann HJ, Hellings PW, Arnavielhe S, Bachert C, Bedbrook A et al. MACVIA clinical decision algorithm in adolescents and adults with allergic rhinitis. J Allergy Clin Immunol. 2016;138(2):367-374.
45. Abelson MB, Shetty S, Korchak M, Butrus SI, Smith LM. Advances in pharmacotherapy for allergic conjunctivitis. Expert Opin Pharmacother. 2015;16(8):1219-1231.
46. Venter C, Meyer R. Session 1: Allergic disease: The challenges of managing food hypersensitivity. Proc Nutr Soc. 2010;69(1):11-24.
47. Groetch M, Henry M, Feuling MB, Kim J. Guidance for the nutrition management of gastrointestinal allergy in pediatrics. J Allergy Clin Immunol Pract. 2013;1(4):323-31.
48. Venter C, Groetch M. Nutritional management of food protein-induced enterocolitis syndrome. Curr Opin Allergy Clin Immunol. 2014;14(3):255-62.
49. Nowak-Wegrzyn A, Groetch M. Nutritional aspects and diets in food allergy. Chem Immunol Allergy. 2015;101:209-20.
50. Meyer R, De Koker C, Dziubak R, Venter C, Dominguez-Ortega G, Cutts R, et al. Malnutrition in children with food allergies in the UK. J Hum Nutr Diet. 2014;27(3):227-35.
51. Groetch M, Venter C, Skypala I, Vlieg-Boerstra B, Grimshaw K, Durban R et al. Dietary Therapy and Nutrition Management of Eosinophilic Esophagitis: A Work Group Report of the American Academy of Allergy,Asthma, and Immunology. J Allergy Clin Immunol Pract. 2017;5(2):312-324.
52. Nachshon L, Goldberg MR, Schwartz N, Sinai T, Amitzur-Levy R, Elizur A, et al. Decreased bone mineral density in young adult IgE-mediated cow's milk-allergic patients. J Allergy Clin Immunol. 2014;134(5):1108-13 e3.
53. Kim J, Kwon J, Noh G, Lee SS. The effects of elimination diet on nutritional status in subjects with atopic dermatitis. Nutr Res Pract. 2013;7(6):488-94.
54. Meyer R, De Koker C, Dziubak R, Skrapac AK, Godwin H, Reeve K, et al. A practical approach to vitamin and mineral supplementation in food allergic children. Clin Transl Allergy. 2015;5:11.
55. Fiocchi A, Schunemann HJ, Brozek J, Restani P, Beyer K, Troncone R, et al. Diagnosis and Rationale for Action Against Cow's Milk Allergy (DRACMA): a summary report. J Allergy Clin Immunol. 2010;126(6):1119-28 e12.
56. Luyt D, Ball H, Makwana N, Green MR, Bravin K, Nasser SM, et al. BSACI guideline for the diagnosis and management of cow's milk allergy. Clin Exp Allergy. 2014;44(5):642-72.
57. Epstein TG, Calabria C, Cox LS, Dreborg S. Current Evidence on Safety and Practical Considerations for Administration of Sublingual Allergen Immunotherapy (SLIT) in the United States. J Allergy Clin Immunol Pract. 2017 Jan -Feb;5(1):34-40.
58. Jutel M, Agache I, Bonini S, Burks AW, Calderon M, Canonica W, Cox L, Demoly P, Frew AJ, O'Hehir R, Kleine-Tebbe J, Muraro A, Lack G, Larenas D, Levin M, Nelson H, Pawankar R, Pfaar O, van Ree R, Sampson H, Santos AF, Du Toit G, Werfel T, Gerth van Wijk R, Zhang L, Akdis CA. International consensus on allergy immunotherapy. J Allergy Clin Immunol. 2015 Sep;136(3):556-68.
59. Esch RE, Plunkett GA. Immunotherapy preparation guidelines, rules, and regulation. Curr Allergy Asthma Rep. 2013 Aug;13(4):406-13
60. Calderón MA, Casale T, Cox L, Akdis CA, Burks AW, Nelson HS, Jutel M, Demoly P. Allergen immunotherapy: a new semantic framework from the European Academy of Allergy and Clinical Immunology/American Academy of Allergy, Asthma and Immunology/PRACTALL consensus report. Allergy. 2013 Jul;68(7):825-8.
61. Flokstra-de Blok BM, van der Velde JL, Vlieg-Boerstra BJ, Oude Elberink JN, DunnGalvin A, Hourihane JO, et al. Health-related quality of life of food allergic patients measured with generic and disease-specific questionnaires. Allergy. 2010;65(8):1031-8.
62. Muraro A, Dubois AE, DunnGalvin A, Hourihane JO, de Jong NW, Meyer R, et al. EAACI Food Allergy and Anaphylaxis Guidelines. Food allergy health-related quality of life measures. Allergy. 2014;69(7):845-53.
63. DunnGalvin A, de BlokFlokstra BM, Burks AW, Dubois AE, Hourihane JO. Food allergy QoL questionnaire for children aged 0-12 years: content, construct, and cross-cultural validity. Clin Exp Allergy. 2008;38(6):977-86.
64. DunnGalvin A, Dubois AE, Flokstra-de Blok BM, Hourihane JO. The effects of food allergy on quality of life. Chem Immunol Allergy. 2015;101:235-52.
65. Venter C, Sommer I, Moonesinghe H, Grundy J, Glasbey G, Patil V, et al. Health-related quality of life in children with perceived and diagnosed food hypersensitivity. Pediatr Allergy Immunol. 2015;26(2):126-32.
66. Jutel M, Angier L, Palkonen S, Ryan D, Sheikh A, Smith H, et al. Improving allergy management in the primary care network--a holistic approach. Allergy. 2013;68(11):1362-9.
67. Sackett DL, Rosenberg WM, Gray JA, Haynes RB, Richardson WS,. Evidence based medicine: what it is and what it isn’t. BMJ. 1996;312(7023):71-72
68. Stevens KR. The impact of evidence- based practice in nursing and the next big ideas. Online J Issues Nurs. 2013;19:4
69. Hjorland B. Evidence based Practice: An analysis based on the Philosophy of Science. J Am Soc Info Sci Tech. 2011;62:1301-1310
70. Gastman C. A fundamental ethical approach to nursing: some proposals for ethics education. Nurs. Ethics 2002;9:494-507
71. Cannaerts N, Gastmans C, Dierckx de Castele B. Contribution of ethics education to the ethical competence of nursing students: educators and students’ perception. Nurs. Ethics 2014;21:861-78
72. Dimauro NM Continuous Professional Development.. JCEN 2000;31:59-62

**Appendix I: EAACI Task Force Membership**

The following EAACI members developed these competencies:

| **Name** | **Institution** |
| --- | --- |
| Isabel Skypala (Dietitian)  Chair and principal author  Chair Allied Health IG 2013-2015 | Royal Brompton & Harefield NHS Foundation Trust, London UK |
| Carina Venter (Dietitian) | The David Hide Asthma and Allergy Research Centre, Isle of Wight, UK |
| James Gardner (Nurse) | The Royal Free Hospital |
| Kate Grimshaw (Dietitian)  Chair Allied Health IG 2015-2017 | Southampton Children’s Hospital, Southampton, UK and Southampton University Faculty of Medicine, Southampton, UK |
| Nicolette de Jong (Scientist) | Erasmus MC, Dept of Internal Medicine, Rotterdam, Netherlands |
| Inger Kull (Nurse) | Sachs Children’s and Youths hospital and Karolinska Institutet, Stockholm, Sweden |
| Jan de Mochy (Physician) | UMCG,  [Groningen, Netherlands](https://www.linkedin.com/vsearch/p?f_G=nl%3A5665&trk=prof-0-ovw-location) |
| Dermot Ryan (Physician) | GP, Loughborough. Honorary Clinical Research Fellow, University of Edinburgh |
| Berber Vlieg-Boerstra (Dietitian) | Emma Children's Hospital, Academic Medical Centre, Amsterdam, and Erasmus MC, Dept of Internal Medicine Netherlands |
| Emilia Vassilopoulou (Dietitian) | University of Nicosia, Nicosia, Cyprus |
| Andrew Williams (Nurse) | Guys and St Thomas’ NHS Foundation Trust, London, UK |
